# Subduction age and stress state control on seismicity in the NW Pacific subducting plate

**DOI:** 10.1038/s41598-022-16076-8

**Published:** 2022-07-20

**Authors:** Nicola Alessandro Pino, Vincenzo Convertito, Cataldo Godano, Claudia Piromallo

**Affiliations:** 1 Istituto Nazionale di Geofisica e Vulcanologia, Osservatorio Vesuviano, Via Diocleziano, 328, 80134 Napoli, Italy; 2 Dipartimento di Matematica e Fisica, Università della Campania “Luigi Vanvitelli”, Via Vivaldi, 43, 81100 Caserta, Italy; 3 Istituto Nazionale di Geofisica e Vulcanologia, Sezione di Roma 1, Via di Vigna Murata, 605, 00143 Rome, Italy

**Keywords:** Solid Earth sciences, Seismology

## Abstract

Intermediate depth (70–300 km) and deep (> 300 km) earthquakes have always been puzzling Earth scientists: their occurrence is a paradox, since the ductile behavior of rocks and the high confining pressure with increasing depths would theoretically preclude brittle failure and frictional sliding. The mechanisms proposed to explain deep earthquakes, mainly depending on the subducting plate age and stress state, are generally expressed by single parameters, unsuitable to comprehensively account for differences among distinct subduction zones or within the same slab. We analyze the Kurile and Izu–Bonin intraslab seismicity and detail the Gutenberg–Richter *b*-value along the subducted planes, interpreting its variation in terms of stress state, analogously to what usually done for shallow earthquakes. We demonstrate that, despite the slabs different properties (e.g., lithospheric age, stress state, dehydration rate), in both cases deep earthquakes are restricted to depths characterized by equal age from subduction initiation and are driven by stress regimes affected by the persistence of the metastable olivine wedge.

## Introduction

More than one fifth of the seismic events with magnitude M > 5 are intermediate depth (70–300 km) or deep (> 300 km) earthquakes (e.g., ref.^1^). They occur along variously shaped dipping bands commonly indicated as Wadati–Benioff zone and their location is limited to subduction regions (e.g., ref.^1^). Like shallow events, deeper earthquakes are associated with sliding along shear planes without significant volumetric component^2^, and respect the general frequency-magnitude distribution^3,4^ (Gutenberg–Richter law) and the aftershock time decay^5^ (Omori law). Nevertheless, intermediate and deep earthquakes appear as a paradox with respect to the temperature and pressure conditions at depth, which would imply that rocks rather yield plastically. Thus, a mechanism allowing stress loading and strain release and implying reduction of rock strength, different from brittle-frictional sliding, is required to explain their occurrence.

The global frequency-depth distribution of earthquakes exhibits stable features among different subduction zones, with a general exponential decay with depth, reaching a minimum at ~ 300 km, and a slow increase up to about 500 km followed by a more marked raise to reach a local maximum at ~ 600 km; then the seismicity abruptly decreases to zero at ~ 680 km (e.g., ref.^6^). This bimodal behavior suggests that the conditions of stress load and release are not homogeneously distributed with depth in the subducting plates. Several processes have been proposed for originating events deeper than 70 km, with dehydration embrittlement^7–9^, transformational faulting^10,11^, and thermal runaway^12,13^ representing the most likely source mechanisms^1,6^. These are generally considered to occur alternatively, with dehydration embrittlement considered as the viable mechanism mainly for depth less than ~ 300 km, while the latter two as active for larger depths and both predicted to occur in presence of the metastable olivine wedge (MOW). The MOW is the wedge within the coldest core of slabs where olivine transformation into a higher density form is delayed, due to kinetic effects, and metastable olivine may persist deeper than 410 km. Each mechanism has difficulties in explaining all the lines of evidence by itself and the concurrence of more than one of them seems to be possible^14^, though.

Each of these processes is supposed to be active in a depth range depending on the water content and on the thermal state of the subducting plate. The thermal state, in turn, is determined by age and sinking velocity of the slab, and by the time from subduction initiation: the older and faster the subducting plate, the deeper the dehydration and the phase transformations within the slab, thus increasing the depth at which the conditions for shear sliding may exist. The maximum earthquake depth is generally considered to increase among subduction zones as a function of the thermal parameter ϕ = *A*_*L*_*∙V*_*Z*_, where *A*_*L*_ and *V*_*Z*_ indicate respectively the age of the subducting lithosphere and the slab’s sink rate, i.e., the vertical component of the subduction velocity^11^.

Whatever the physical process, the source mechanisms act in response to a stress varying with depth. Typically, (1) prevalent down-dip extension is observed for intermediate depth earthquakes in slabs with no deep events or with clear seismicity gaps between deep and intermediate-depth events, (2) down-dip compression is nearly always associated with deep events, and (3) slabs with continuous seismicity down to the base of the upper mantle might exhibit compression throughout their entire length^15^. However, detailed analyses of the intermediate depth seismicity reveal that, in most subduction zones, earthquakes form a double seismic zone along two planes parallel to the slab surface, in some cases characterized by down-dip compression and down-dip extension respectively on the upper and lower planes^16^.

Overall, the stress in the subducting plate is originated by its negative buoyancy, by viscous resistance from the upper (e.g., ref.^17^) and the lower mantle^18,19^, and possibly by additional forces due to density^18,20,21^ and/or volume^22–24^ changes associated with phase minerals’ transformations within the slab. Bending and unbending of the subducting plane are also invoked as primary source of stress^21^.

Among distinct subduction zones there are significant differences in the depth range where seismicity occurs and considerable lateral variations are present within the same slab^25^—especially deeper than ~ 300 km (ref.^26^)—evidencing that the slab thermal and stress’ states may change laterally over relatively short distances. These slab characteristics cannot be estimated directly and their assessment requires geodynamic modelling, relying on the knowledge of the slab’s lithosphere age and the time of its subduction (e.g., refs.^20,21^). Insight on the stress state can also be derived from estimates of the earthquakes’ stress drop, which is correlated to the differential stress^27^. However, the stress drop estimate strongly depends on the adopted source model and is generally affected by considerable uncertainty^28^. Thus, the value associated with the seismicity in a certain area is usually derived by averaging the results from a relatively large number of earthquakes (e.g., refs.^29,30^), making the stress drop not suitable to map small-scale spatial variations of the stress state.

On the other hand, laboratory experiments and the analysis of crustal earthquakes reveal that the differential stress is also (inversely) correlated to the *b*-value (e.g., refs.^27,31^) of the Gutenberg–Richter (G–R) frequency-magnitude relation^32^ log(N) = *a* + *b*M, with *a* and *b* constants, and N indicating the number of earthquakes with magnitude ≥ M. The *b*-value characterizes the relative abundance of small compared to large earthquakes, and is commonly used to derive images of the differential stress acting in a volume, providing meaningful results on extended length-scale ranges—from acoustic emissions in laboratory experiments (e.g., refs.^33,34^) to large crustal earthquakes (e.g., refs.^35,36^)—with low and high *b*-values representing respectively high and low differential stress areas. The robustness of these results confirms the confident use of the *b*-value as a stress meter and to map the time/space variations of stress in these domains (e.g., ref.^37^). Whether this can be appropriate also for deeper earthquakes is still unclear.

Intermediate and deep earthquakes as well are known to follow the Gutenberg–Richter statistics, with significant regional variations of the *b*-value—larger than what observed for shallow events—ranging from ~ 0.4 to ~ 1.2 (ref.^38^). Moreover, although occurring with mechanisms different from frictional faulting, these events exhibit a double-couple mechanism, indicating absence of volumetric component and slip on a planar surface^1,39^.

In general, analyzing earthquakes by means of macroscopic parameters, such as magnitude, stress drop, seismic energy, average dislocation, and rupture area, gives the chance to investigate the frictional conditions independently of the fracture process^40^. In this regard, the results of massive analyses of intermediate and deep earthquakes demonstrate that, in spite of the possibly different physics, deep and shallow earthquakes display similarities for most of the seismological observables, such that “deep and shallow earthquakes differ little even in key aspects of the rupture process” (ref.^39^). Thus, whatever the faulting mechanism, given adequate elastic strain energy to generate instability, the larger the available differential stress the more it can grow into a large fracture. This means that a higher stress increases the probability of larger fractures with respect to small ones, resulting in lower *b*-value.

The evidences that the rupture process of deep and shallow earthquakes does not reveal appreciable diversity imply that, if distinct mechanisms must be invoked to explain the occurrence of earthquakes in different depth ranges, this must affect only imperceptibly the resulting earthquake source properties^39^. This leads to conclude that a single relation between differential stress and *b*-value should hold for earthquakes at any depth.

Previous analyses estimated a single value for vertical sections of each study region (e.g., ref.^41^), or separated intermediate and deep events (e.g., ref.^14^), or focused just on intermediate events (e.g., ref.^42,43^). A few studies investigated the spatial variation of the *b*-value along vertical cross sections^44^ or along the slab surface^45^, but limiting to the upper 150–200 km depth. A comparison of the relative intensity of the stress within slabs predicted by theoretical models with estimates from earthquake data from the whole upper mantle depth range is therefore still lacking.

Here, in order to investigate the possible relationship between the characteristics of the subducting lithosphere and the mechanism generating the stress accumulation for intermediate and deep earthquakes, we analyze several features of the Kurile (*K*) and Izu–Bonin (*I–B*) subduction zones along with the earthquakes’ location and frequency-magnitude distribution. These two subduction zones are chosen as they represent distinct regions of the same subducting oceanic plate and are characterized by similar lithosphere thickness^46,47^ and similar subduction stage, with horizontally lying slab at the 660 km discontinuity^48^, as testified by abundant geophysical data. Furthermore, both are interested by deep seismicity possibly connected with the persistence below the 410 km phase transition of the MOW, as predicted by thermal models^49^ and detected by analyses of the seismic waves’ propagation through the slab^50^ and seismic tomography^51^. On the other hand, the two slabs have different properties (e.g., different lithospheric age, stress state, and dehydration rate). We develop 2D *b*-value maps throughout the extension of the subducted planes and discuss them in terms of heterogeneous differential stress, related to the slab kinematics and dynamics, explaining the observed distribution and dynamic regime of deep focus earthquakes.

## Results

### Intraslab seismicity distributions versus subduction related parameters

**Figure 1:**
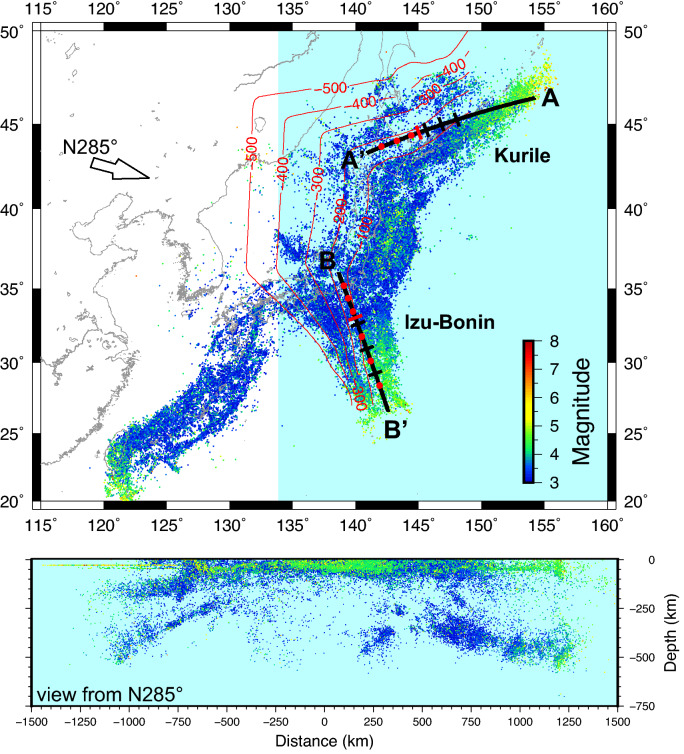
Map of the earthquakes in the northwestern Pacific subduction zone. Only events with M ≥ 3.0 are displayed, which are better located and more representative of the stress state in the slab. The labeled red lines depict depth contours of the upper boundary of the subducting slab as estimated from earthquakes’ location^52^. The seismic events in the light blue shaded box, as seen from N285° (arrow on the map), are reported in the vertical cross-section at the bottom. The black lines indicate the trace of the Kurile and Izu–Bonin vertical sections shown in Figs. 2 and 3, with red ticks marking the origin of the distance axis. The location of the profiles in Fig. 4 (black ticks) and in Supplementary Figs. S3 and S4 (red dots) is also shown.

We used for our analysis the hypocenter locations of earthquakes occurred in the Northwest Pacific subduction zone from 1 January 1998 to 31 March 2016, provided by the Japan Meteorological Agency (JMA) (Fig. 1). The intraslab earthquakes in *K* and *I–B* display an approximately similar bimodal pattern with depth, with a shallower band of seismicity occurring at depth less than 150–200 km all along the trench and deep earthquakes located at increasing depth moving respectively northeastward and southward in the two areas (Fig. 1). Remarkably, the depth extent of the deep seismicity band is almost double in *I–B* with respect to *K*. In between the two bands seismicity is scanty and diffuse.

We assume the data from a recent paleo-reconstruction of the subducting Pacific plate beneath East Asia^52^, derived from a P-wave tomographic model and paleo-age data of ancient seafloor, and analyze the potential connection of the earthquakes’ distribution pattern with some subduction related parameters. We first analyze the potential connection of the earthquakes’ distribution with the thermal parameter ϕ of the subducted plate, along vertical cross-sections, also investigating the relationship with the two factors^11^ contributing to ϕ—the age of the subducting lithosphere *A*_*L*_ (i.e., the period from the birth of the oceanic lithosphere at the mid-ocean ridge to the present) and its sink rate *V*_*Z*_—and with the subduction age *A*_*S*_ (i.e., the time period from the plate subduction at the trench to the present). The results indicate that in both the considered regions, there is no obvious correlation of the spatial distribution of earthquakes with the thermal parameter (Supplementary Fig. S1). Although representing an overall reasonable description of the general thermal state of a subducting plate, it appears to be too rough an estimate for the interpretation of local variations of seismicity distribution along a single slab. For both *K* and *I–B* the gradient of ϕ is oblique or almost perpendicular to what would be expected for the earthquakes’ maximum depth to depend on this parameter. This derives from the distribution of the lithosphere age and the sink velocity (Supplementary Fig. S1), with the latter controlling the product because of its higher relative variation. Both intermediate and deep seismicity bands appear to deepen almost perpendicularly to the equal sink velocity lines.

**Figure 2:**
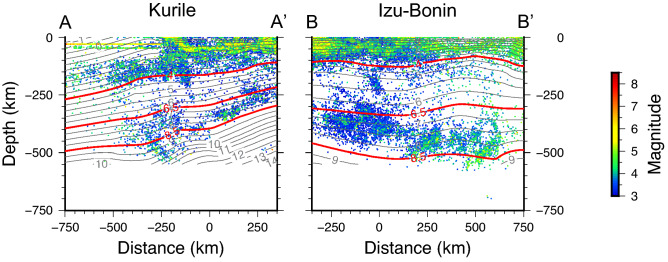
Vertical sections of the seismicity, along the profiles reported in Fig. 1. The profiles are parallel to the trench of the Kurile and Izu–Bonin subduction zones. The lines of equal age from the start of subduction at the trench are also displayed^52^. Apparently, in Izu–Bonin the slab reaches significantly larger depths in the same time span (e.g., 8.5 Ma), implying larger average sink velocity. The intermediate earthquakes are mainly localized at depths characterized by slab age younger than 4 Ma, while the deep events are prevalently enclosed within 6.5 Ma and 8.5 Ma.

For both *K* and *I–B* slabs we observe a close relation between the seismicity depth distribution and the subduction age, with the shallower seismicity band extending above the 4 Ma age line and the deeper one (> 300 km) enclosed between 6.5 and 8.5 Ma age lines (Fig. 2). Note that the distance in depth between the 6.5 and 8.5 Ma lines is almost double in *I–B* with respect to *K*, due to higher sink rate of *I–B* slab.

### Slab-scale maps of the b-value

**Figure 3:**
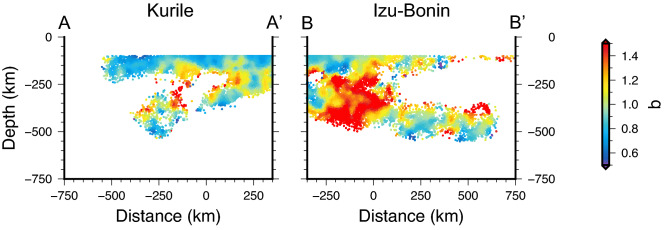
Distribution of the Gutenberg–Richter *b*-value. Estimates for earthquakes in the Kurile and Izu–Bonin subduction zones projected on the vertical sections corresponding to the black traces in Fig. 1. The *b*-value is inversely correlated with the deviatoric stress, thus lower (higher) values indicate higher (lower) stress.

Next, we estimate the G–R frequency-magnitude relation *b*-value (see Methods), which can be used as a proxy to map the state of stress throughout the slab extent, being inversely dependent on differential stress. Analogously to the events’ location pattern, the spatial distribution of *b* for the two considered subduction zones evidences general analogies and significant differences (Fig. 3).

**Figure 4:**
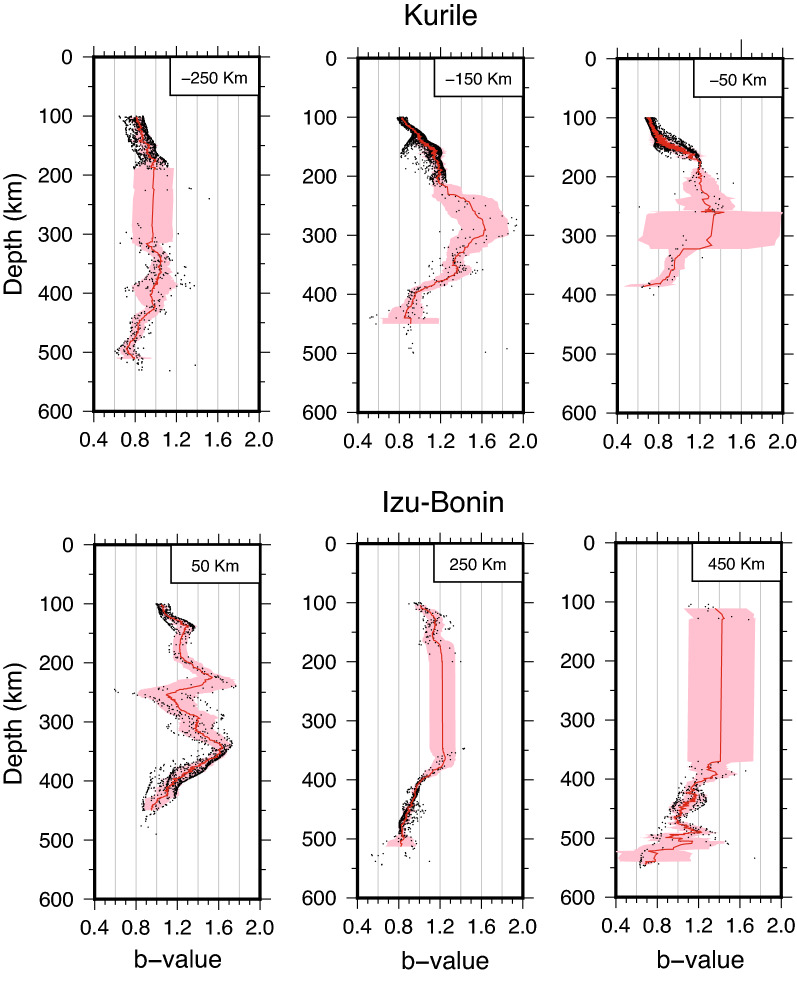
Depth distributions of the estimated *b*-values along selected profiles. Each depth profile includes values along 100 km-thick slices, centered at the distance indicated on top, along the relevant profile. The red lines represent the 30 pts. moving average, except for the 450 km *I–B* profile, for which a 10 pts. average is applied due to the lower number of points available, while the red shaded bands represent the standard deviation of the moving average. See Figs. S3 and S4 for all the profiles.

Lower *b*-values (< 1) result around 100 km depth and at the bottom of the bands of deep seismicity, whereas the intermediate seismicity exhibits relatively high *b* (> 1), in particular where the seismicity distribution is more homogeneous and continuous with depths, with almost no gap between intermediate and deep earthquakes. Actually, at the northern end of *I–B*—where the deep earthquake band is shallower—the seismicity exhibits higher *b* in the whole depth range (~ 200 to ~ 450 km). The whole picture highlights the variability of *b* over the subducting plane and the importance of mapping its heterogeneous distribution with some detail, rather than expressing the frequency-magnitude earthquakes’ distribution either with a single value for the whole slab or by separately grouping intermediate and deep events. To better appreciate the details of the *b*-value spatial variation, we plot in Fig. 4 the distribution with depth of the *b*-value along three selected vertical 100 km-large slices perpendicular to the two sections, i.e., perpendicular to the trench (the complete set of slices is available in Supplementary Figs. S3–S4).

## Discussion

In spite of the different stress regime, and also different average lithosphere age (Supplementary Fig. S1) and dehydration rate, with *K* H_2_O flux double with respect to *I–B* (ref.^53^), the earthquakes’ spatial distributions along the two subducting planes display analogous features, with the two bands of seismicity enclosed by the same subduction age lines (Fig. 2). This evidence is consistent with the hypothesis of different physical processes allowing intermediate and deep earthquakes. It also indicates that the conditions for sliding to take place along shear planes within slabs primarily depend on the time elapsed from the plate subduction at the trench to the present and apparently are not affected by small differences in the lithosphere age. This is because the coarse relation between the depth of earthquakes’ occurrence and the thermal parameter^1^ is mainly controlled by the sinking rate^25^ (the average sink rate is the depth from the trench multiplied by the inverse of the subduction age), which contributes to induce more deformation within the slab. It is possible that even the time to the thermal equilibrium with the surrounding mantle has a stronger dependence on the average sink velocity than the age of the subducted lithosphere.

In order to discuss the relative stress conditions along the subduction zone with respect to the slab kinematics, useful information on the direction of the prevalent stress acting within the slabs can be deduced from the focal mechanisms, by considering the direction of the compressional (P) axes from the centroid moment tensor solutions^54^ (gCMT) (Supplementary Fig. S2). At depth between 100 and 200 km the *K* earthquakes are associated with clear tensional stress in the south (distance > − 350 km along the profile) and compressional stress parallel to the slab velocity (i.e., the gradient of the lines of equal slab age) around the northern end of the profile (distance < − 350 km), where the slab is also characterized by steeper subduction angle^47^, larger sink velocity, and slightly younger lithosphere age (Supplementary Fig. S2). Instead, in-plane compression is associated with all the *K* deep events^54^ (depth > 300 km) (Supplementary Fig. S2). A different situation results in *I–B*, with in-plane compression along the whole slab and the entire depth range^54^. It is worth noting that, either in *K* or *I–B*, the P axis of the deep events is not necessarily downdip; at depths larger than 300 km the compression is perpendicular to the lines of equal sink velocity, i.e., parallel to the direction of motion of the slab, which significantly differs from the trench-perpendicular direction in both areas.

**Figure 5:**
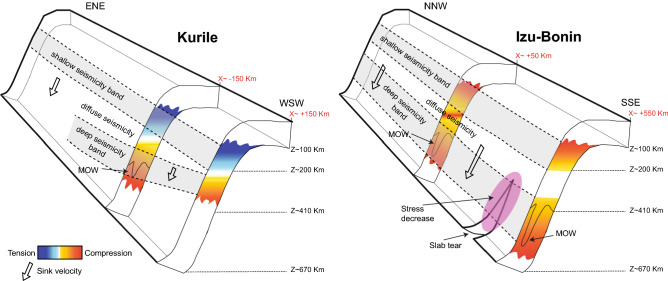
Sketch summarizing the features observed in the two subduction zones. The shallow and deep seismicity bands are drawn as gray stripes and are separated by the area of diffuse seismicity. The stress distribution with depth is schematically drawn along two cross sections for each subduction zone, with blue shading for tensional and red shading for compressional stress, darker colors meaning larger absolute values. The distance of the cross section in km along the profiles, according to Figs. 2, 3, S3, S4, is marked in red. The black wedge-shaped line at about 410 km depth represents the Metastable Olivine Wedge (MOW). The pink oval shape in Izu–Bonin roughly indicates the area of stress decrease due to the presence of a slab tear.

Interpreting the distribution of the *b*-value in terms of stress and slab kinematics, the two subductions evidence general common trends with depth, with shorter scale differences indicating a complex state of stress (Figs. 3, 4, and Supplementary Figs. S3, S4). All the *K* slices indicate decreasing stress trend from 100 km to ~ 200 km depth (Figs. 4 and Supplementary Fig. S3); between 200 and 350 km depth, the seismicity is quite sparse (except for the southern end of the profile) and the deviatoric stress, when appraisable, reaches its lowest value. Below this depth range the stress increases all the way down to the bottom of the seismicity. By considering the local direction of the principal compressional axis, as deduced from the focal mechanisms, we conclude that the *K* subduction zone is associated with tensional stress regime decreasing with depth in the shallow upper mantle, then the stress changes to compressional, increasing while approaching the 670 km discontinuity (Fig. 5). This picture is consistent with the theoretical predictions for a slab sinking in the viscous upper mantle, characterized by phase transitions at 410 km and 670 km depth (representing major density and/or viscosity changes), and subject to stress prevalently arising from buoyancy forces (e.g., refs.^18,20^). In this framework, the lower stress deduced for depths between 200 and 350 km—associated with the onset of the compressional regime^54^ (Supplementary Fig. S2) —derives from the presence of the MOW below the 410 km discontinuity (Fig. 5), representing a buoyancy anomaly that produces a “parachute effect”^11^ hindering the sink. This would reduce the in-plane tension and even invert the stress, giving rise to compression as observed.

At *I–B*, above 200 km the stress diminishes with depth as well, although the trend is less clear in the southern slices (distances > 150 km along the profile in Fig. S4), likely because of the lower detection capability of seismic networks in this offshore area. However, contrarily to what observed at *K*, at *I–B* the stress in the shallow upper mantle is compressional^54^ (Supplementary Fig. S2). We suggest that this difference could result from the higher *I–B* average sink velocity (Fig. 2), which induces in-plane compression along the entire slab length (Fig. 5). Also, a larger viscous resistance of the surrounding mantle, whose flow is horizontal and mostly parallel to the trench at least above 370 km depth^55^, may enhance the compressive stress above 200 km. The local stress increase between 200 and 350 km depth, corresponding to the *b* relative minimum in the northern *I–B* slices (Fig. 4 and Supplementary Fig. S3), derives from the positive buoyancy anomaly of the MOW (Fig. 5), which resists the slab sinking and boosts the in-plane compression.

In the framework of our interpretation, the high *b*-values in the depth range of 200 to 450 km and in the distance range of − 300 to 100 km (Figs. 3, 4, and Supplementary Fig. S4) should represent a remarkably low stress area. We suggest that this could be associated with the older and faster subducting lithosphere in *I–B* (Figs. 2 and Supplementary Fig. S1). Indeed, these characteristics allow the slab to retain a larger amount of water and take this to relatively large depths (e.g., ref.^56^)—significantly larger than what expected for the Kurile slab—as also demonstrated by finite element modeling of mineralogically bound water in downgoing slabs^53^. The larger water content would dramatically drop the yield stress in the slab, as well known to occur for hydrous olivine at mantle pressure/temperature conditions^57^.

Analogously to *K*, below ~ 350 km depth the stress increases again due to the resistance to sink exerted by the 670 km discontinuity, except for the slices at x = 350 km and x = 450 km (Fig. 4 and Supplementary Fig. S4), where the general increase is superimposed by localized lower stress around 500 km depth. This latter is located where a slab vertical tear (Fig. 5) has been detected^58^ and earthquakes exhibit lateral tension mechanism^59^. The slab tear reduces or possibly cancels the compression produced by the slab sinking and rotates the stress horizontally.

The *b*-value map that we obtain is limited to areas where the analysis is feasible according to the seismicity distribution and to the applied methodology and settings (specified in Methods). Consequently, the derived (qualitative) stress mapping cannot cover the entire slab and some areas are necessarily overlooked (white areas in Fig. 3). In the covered areas the low uncertainties in the *b*-value estimates, as attested by standard errors (Supplementary Fig. S5), ensure that variations of the resulting *b*-value are well significant. Similarly, the relatively low values of the completeness magnitude (Supplementary Fig. S5) ensure the robustness of the resulting *b*-values.

If a single relation between differential stress and *b*-value must hold for earthquakes at any depth, it should be possible to extrapolate the relation deduced from shallow earthquakes and get an order of magnitude for the differential stress acting on intermediate and deep seismicity. Thus, we checked the implications of this conclusion and tentatively used the relation *b* = 1.23–0.0012(*σ*_*1*_–*σ*_*3*_) (ref.^31^), with the stress in MPa. The results indicate differential stress values within the slab up to hundreds of MPa, comparable to what predicted at those depths by thermomechanical numerical simulations of the subducting lithosphere (e.g., ref.^60^) and to intermediate and deep earthquakes stress drop estimates (up to hundreds of MPa; e.g., ref.^39,40^), which should be considered as a lower bound for the differential stress.

Finally, our analysis allows imaging the stress conditions throughout the slab extent, highlighting its strong heterogeneity and suggesting a thorough perspective to look at the origin of deep focus earthquakes. The Gutenberg–Richter *b*-value computed from the Kurile and Izu–Bonin intraslab seismicity is used as a proxy to detail the stress state along the subducted planes. Despite the slabs’ different properties, in both regions the deep earthquakes are restricted to depths characterized by equal age from subduction initiation and are driven by stress regimes affected by the persistence of the metastable olivine wedge. Altogether, the results obtained for the two investigated areas can be explained in terms of stress generated by the buoyancy forces—mainly affected by the slab sink velocity and the mineralogical phase transitions—without the need to invoke bending/unbending of the subducting plane, whose associated stress would exhibit a significantly different trend with depth^21^.

## Methods

### Gutenberg–Richter b-value determination

The dataset for *b*-value determination is the JMA earthquake hypocenters catalog for the Northwest Pacific subduction zone (1 January 1998 to 31 March 2016) of Fig. 1. Being interested in the genesis of the intraslab earthquakes, we exclude events shallower than 100 km, introducing a sharp cut off of crustal and/or upper plate seismicity. We obtained cross-section *b*-value imaging by implementing the Distance Exponential Weighted (DEW) method^61^. Compared to the traditional approaches (e.g., ref.^62^) based on the gridding of the investigated area, the DEW does not use the closest N events to a grid node nor it considers all events from inside a constant search radius *R* centered on the node. Instead, the DEW method assigns each earthquake a distance-dependent weight. In the applications to this study the weights are assigned depending on the distance-dependent decay function w(d) = λe^−λd^, being d the distance and λ a decay parameter that must be set on the basis of the specific application. Once the weights have been obtained for all the earthquakes, we calculated the *b*-value by using the maximum likelihood approach^63^ and computing the weighted mean magnitude value. The standard error has been obtained by using the Shi and Bolt formula^64^. The minimum magnitude of completeness (M_c_), which defines the catalogue threshold for determination of the *b*-value for each node, is computed by using the maximum curvature technique (e.g., ref.^63^).

The investigated areas are gridded by 2 km-spaced nodes along both the horizontal distance and the depth. After performing several tests, we set the parameters that allow obtaining a good compromise between obtaining stable *b*-value features and a good coverage: for each grid point, we require at least 100 events above a threshold magnitude value (set to 3) inside a circle whose radius is set to 75 km. Next, once computed M_c_, we require a minimum of 50 events with magnitude larger than M_c_ to compute *b*. We use the decay function w(d) = 0.7e^−0.07d^ (ref.^60^). The *b*-value map obtained following this methodology are shown in Fig. 3.

In Supplementary Fig. S5 the distribution of the minimum magnitude of completeness M_c_ and of the standard error $\sigma$ are shown. The M_c_ in *K* slightly increases (by roughly 0.5) at the edge of the shallow seismicity band and for distances < − 250 km in the deep seismicity band. The M_c_ increase in *I–B* is larger (about 1.0) both at surface and depth for distances larger than 350 km. In both cases this is due to the reduced detection capability of the seismic networks in these off-shore regions. The standard errors in both *K* and *I–B* are on average very close to zero (Supplementary Fig. S5), with higher values along the edge of the distribution, where the earthquakes are fewer and the *b*-value estimate is less robust.

It is worth noting that JMA ranks the resulting locations into high-precision (H-P) and low-precision (L-P) hypocenters. For earthquakes in the Kurile area they declare epicenter error of less than 10′ (~ 18 km) and between 10′ (~ 18 km) and 15′ (~ 25 km), respectively for the two categories. About half of these quantities (5′ for H-P; between 5′ and 10′ for L-P) are associated to locations in the remaining areas. The error associated with the depth is always lower than 10 km. In our analysis, about 95% and more than 96% earthquakes are high-precisely located events, respectively for Kurile and Izu–Bonin. Moreover, when considering only the events with magnitude above M_c_, these amounts obviously increase, being stronger earthquakes better located than small ones. Thus, the location uncertainty is expected to cause only minor effects on our estimates of the *b*-value.

## Supplementary Information

Supplementary Figures.

## Data Availability

The earthquake location data are available from the Japan Meteorological Agency (JMA; https://www.data.jma.go.jp/svd/eqev/data/bulletin/hypo_e.html).
