# Supplementary material for: Subduction age and stress state control on seismicity in the NW Pacific subducting plate
Source: Sci Rep. 2022 Jul 20;12:12440. doi: 10.1038/s41598-022-16076-8 (PMC9300704; doi:10.1038/s41598-022-16076-8)
Supplement: Supplementary file 1 — Supplementary Figures. [file 41598_2022_16076_MOESM1_ESM.docx]

Supplementary Information

Subduction age and stress state control on seismicity in the NW Pacific subducting plate

Nicola Alessandro Pino^1^*, Vincenzo Convertito^1^, Cataldo Godano^2^ & Claudia Piromallo^3^**^†^**

^1^Istituto Nazionale di Geofisica e Vulcanologia, Osservatorio Vesuviano, Via Diocleziano, 328, 80134, Napoli, Italy.

^2^Università della Campania “Luigi Vanvitelli”, Dipartimento di Matematica e Fisica, Via Vivaldi, 43, 81100, Caserta, Italy.

^3^Istituto Nazionale di Geofisica e Vulcanologia, Sezione di Roma 1, Via di Vigna Murata, 605, 00143, Roma, Italy.

**Corresponding authors:**

***e-mail: [alessandro.pino@ingv.it](mailto:alessandro.pino@ingv.it)**

**^†^e-mail: claudia.piromallo@ingv.it**


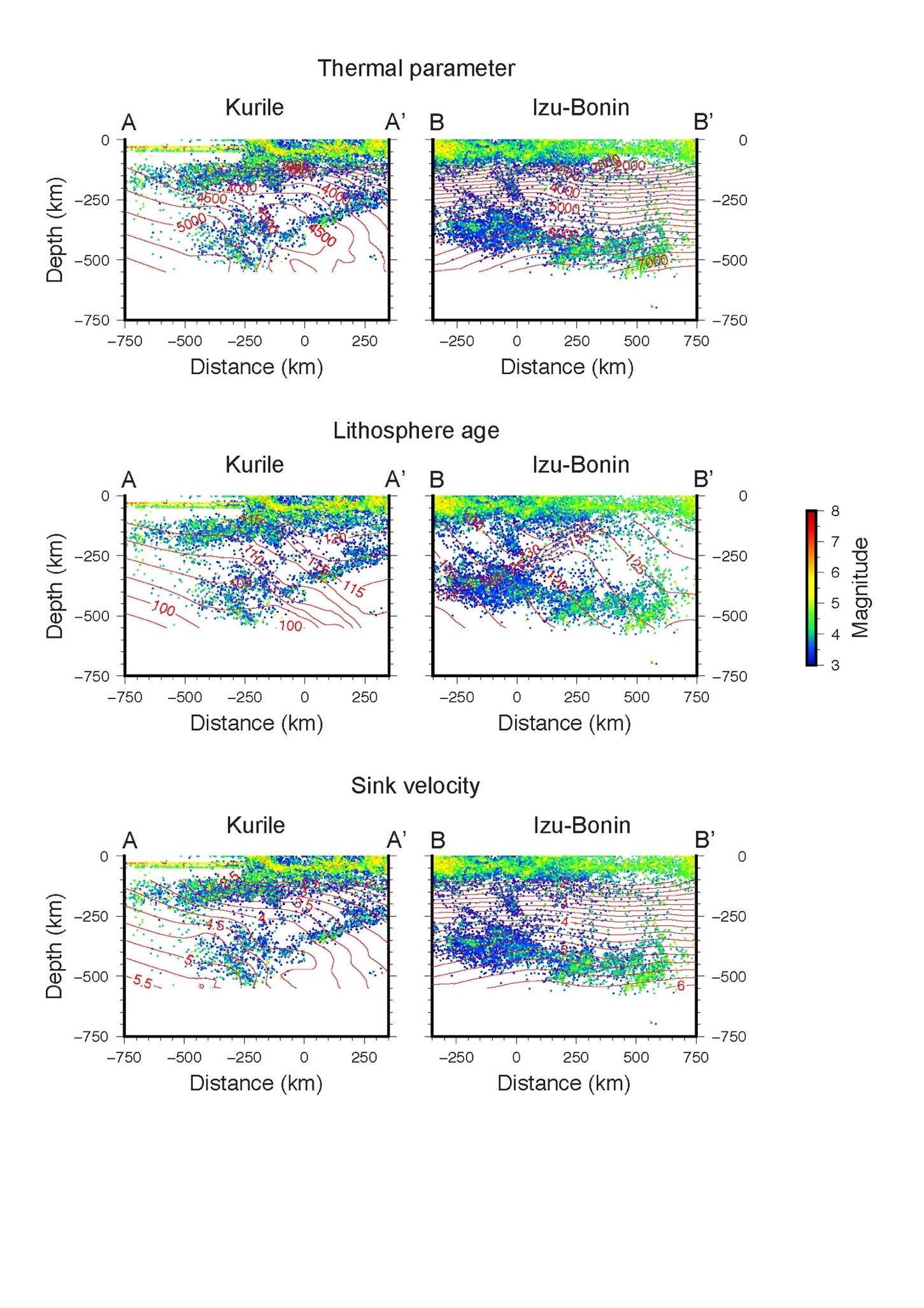


Supplementary Figure S1. Vertical cross-sections of the seismicity, along the two profiles reported in Fig. 1. The profiles are parallel to the trench of the Kurile and Izu-Bonin subduction zones. Data are projected onto distance-depth vertical sections and the values are resampled over a regular grid. The lines of equal thermal parameter (top), lithosphere age (middle), and sink velocity (bottom) are also displayed. The events are ordered in the projection according to their magnitude, with lower magnitude earthquakes behind, to evidence the areas where stronger events occur.


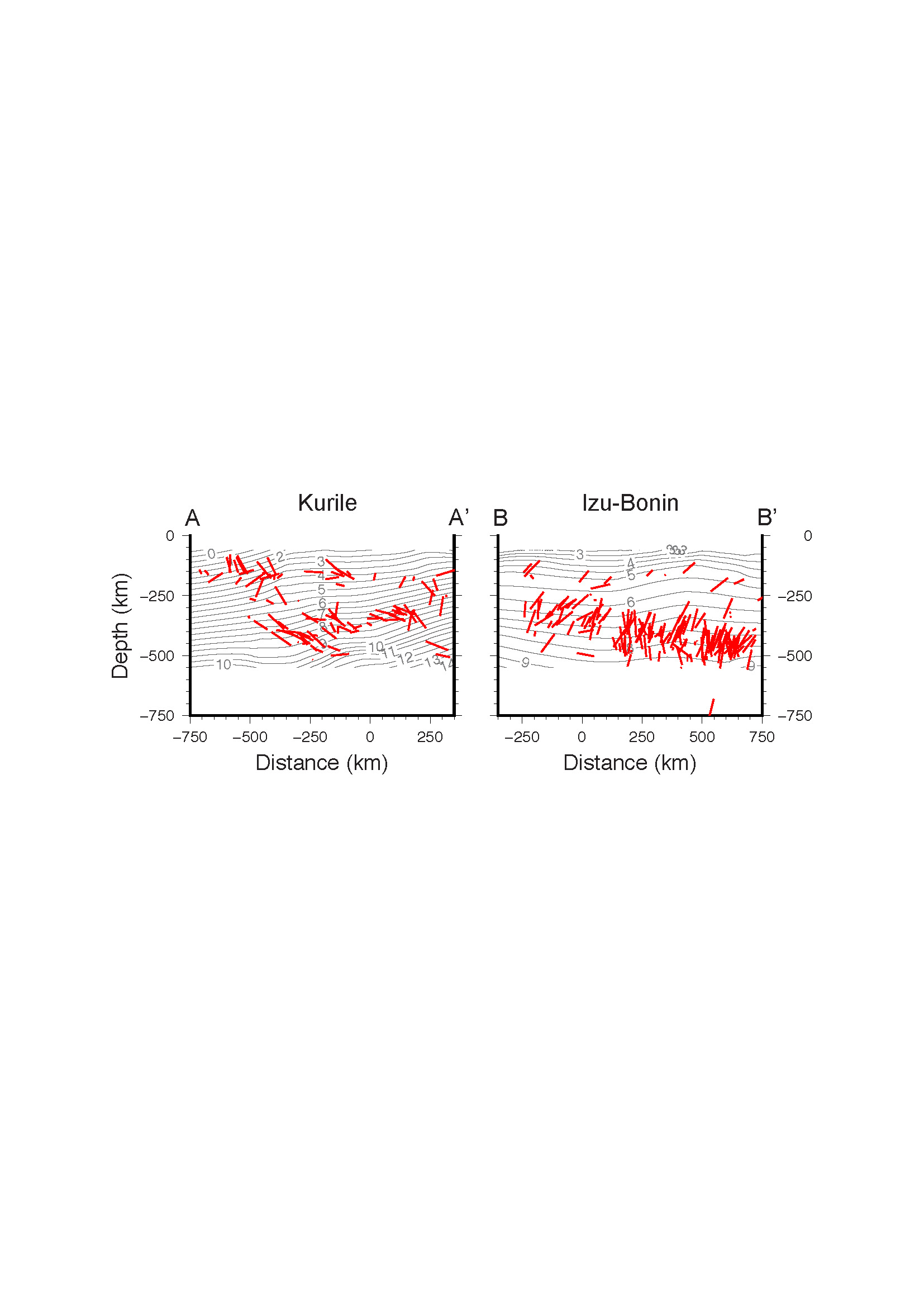


Supplementary Figure S2. *P* axes of the earthquakes’ gCMT focal mechanisms, (https://www.globalcmt.org/CMTsearch.html) projected on the vertical sections corresponding to the profiles indicated in Fig. 1 of the article. The vertical projection is chosen in order to compare the *P* axes with the slab age. With respect to the sink direction (i.e., the gradient of the lines of equal slab age), the stress regime deduced from the principal compression axes for intermediate events in Kurile and Izu-Bonin is respectively tensional and compressional, while in both areas it is compressional for deep earthquakes.

Supplementary Figure S3. Depth distributions of the estimated *b*-values for the Kurile subduction zone. Each depth profile includes values along 100 km-thick slices, centered at the distance indicated on top, along the relevant section. The red lines represent the 30 pts. moving average except for the -50 km profile, for which a 10 pts. average is applied due to the lower number of points available, while the red shaded bands represent the standard deviation of the moving average.

Supplementary Figure S4. Depth distributions of the estimated *b*-values for the Izu-Bonin subduction zone. Each depth profile includes values along 100 km-thick slices, centered at the distance indicated on top, along the relevant section. The red lines represent the 30 pts. moving average, except for the 450 km and 550 km profiles, for which a 10 pts. average is applied due to the lower number of points available, while the red shaded bands represent the standard deviation of the moving average.

Supplementary Figure S5. Distribution of the minimum magnitude of completeness M_c_ used for determination of the *b*-value (top), and of the *b*-value standard error $\boldsymbol{\sigma}$ (bottom), for the two subduction zones. See Methods section for details.
